# Beyond criterion: cognitive flexibility in wild striated caracaras

**DOI:** 10.1098/rsbl.2025.0495

**Published:** 2025-11-19

**Authors:** Katie J. Harrington, Megan L. Lambert

**Affiliations:** ^1^ Messerli Research Institute, Department of Interdisciplinary Life Sciences, University of Veterinary Medicine Vienna, Vienna, Austria

**Keywords:** physical cognition, learning curve analysis, trials-to-criterion, reversal learning, interindividual variation, cognition in the wild, replicability

## Abstract

Cognitive flexibility, the capacity to adapt to changing conditions, is often assessed with reversal learning, in which a learned association must be updated after reward contingencies change. Trials-to-criterion (TTC) is a widely applied learning threshold, but it can misrepresent performance; some individuals improve steadily but fail to reach the criterion due to variability (false negatives), while others meet it through a spike without sustained learning (false positives). We evaluate TTC limitations and demonstrate learning curve analysis as a more nuanced approach to investigate learning dynamics. We tested wild striated caracaras (*Phalcoboenus australis*) using a two-choice discrimination task followed by a reversal task and compared TTC with trial-level modelling. Although the group showed overall improvement, individual trajectories varied widely. TTC both over- and underestimated learning, misclassifying inconsistent performers and overlooking gradual improvers. In contrast, learning curves captured trajectory, stability and consistency of change. We argue that continued reliance on binary thresholds obscures the dynamics of learning, and that slope- and trajectory-informed analyses provide a more accurate and ecologically valid framework for assessing learning in the wild.

## 1. Introduction

In a rapidly changing world, the ability to adjust behaviour to novel or shifting circumstances is critical for survival and reproduction [1–3]. This adaptability, broadly termed behavioural flexibility, includes cognitive processes such as learning and inhibition, and behavioural traits like neotic style, motivation and exploration [1] (but see [4–6]). Cognitive flexibility, a core component, refers to the ability to modify previously learnt associations or strategies in light of new information [7].

Reversal learning (RL) tasks are widely used to assess cognitive flexibility by measuring how individuals adapt to changing stimulus–reward contingencies [7,8]. Trials-to-criterion (TTC), the number of trials needed to reach a predefined accuracy, is a commonly applied learning threshold [9], following the logic that, for example, 10 correct out of 12 consecutive trials is unlikely to occur by chance (binomial test, *p* = 0.019). However, this logic is flawed, as it applies to a single block of 12 trials, whereas TTC applies a sequentially moving window until the criterion is met. This repeated testing inflates false positives, and the effect compounds as trial number increases. To illustrate the problem, we simulated 10 000 random agents (*p* = 0.5), applying a 12-trial sliding window to detect runs of ≥10 correct responses. False positives rose from approximately 10% after 25 trials to >30% after 75, demonstrating how Type I error scales with opportunity in binary choice tasks. Despite long-standing and ongoing critiques [10–13], TTC remains widely used [9].

TTC moreover reduces complex behaviour to a snapshot, which obscures meaningful dynamics and risks further misclassifying individuals. For instance, gradual improvers may fail to reach the criterion due to variability (false negatives), while others may hit the criterion through a performance spike without sustained learning (false positives). These issues are amplified in naturalistic settings, where individuals’ availability, motivation, social rank and context affect access to test apparatuses and result in uneven participation, with some individuals completing many trials and others dropping out [13–15], and where the biological relevance of when learning occurs also varies with species’ ecology and life history—what seems protracted in a short-lived animal may be negligible in a long-lived one. This variability underscores the need for trajectory-based alternatives to TTC as interest in cognitive variation under ecologically valid conditions grows [13,15–19].

In response, approaches have been proposed that model trial-wise performance to directly test for systematic improvement. In binary designs, comparing estimated learning slopes to null distributions from simulated random agents provides a process-based measure that reduces bias from thresholds whose interpretive value declines in extended, autocorrelated trial sequences [20,21]. Comparable approaches have been adopted in multi-choice reversal tasks, where error-based estimates capture learning trajectories without reliance on arbitrary thresholds [22,23], underscoring a shared movement across task types towards more process-sensitive analyses.

Cognitive flexibility has been extensively studied in captivity, and increasingly, in wild populations [24–27], which is essential for understanding how individual differences are shaped by experience, environment and evolutionary pressures [13,15,18]. Striated caracaras (*Phalcoboenus australis*) on the Falkland (Malvinas) Islands provide a compelling wild model. These inquisitive, social falcons face strong seasonal shifts in resource availability [28,29], and as broad-spectrum foragers—scavenging, hunting and exploiting natural and anthropogenic resources—exemplify an ‘open-programme’ species capable of adapting behaviour to diverse opportunities [30,31]. Prior work has documented their neophilia, foraging innovation and problem-solving abilities [32–35]. Their high motivation, tolerance of humans and tendency towards repeated, effortful foraging [35] make them ideal for evaluating TTC limitations and illustrating how learning curves reveal more nuanced patterns of behavioural change.

Here, we adapted the RL paradigm to assess cognitive flexibility in wild striated caracaras, measuring individuals’ ability to form and reverse a novel colour-reward association when contingencies changed. To capture both population trends and individual variation, we modelled trial-level success, then analysed learning slopes against null distributions, providing a process-based alternative to TTC. Our results shed light on the cognitive ecology of this behaviourally divergent falcon lineage [36], and demonstrate the advantages of trajectory-informed analyses for assessing flexibility in the wild.

## 2. Methods

### (a) Study site and species

We tested wild striated caracaras from 17 August to 11 September 2024 (austral winter) at the only settlement on privately owned Saunders Island, Falkland Islands (51.3667° S, 60.0833° W). The site is part of a long-term monitoring project; unmarked birds were ringed and weighed prior to testing (for methods, see [33]). Two birds participated in previous, distinctive cognitive behavioural experiments [33,37]. Voluntary participation may have elicited a STRANGE bias (e.g. towards bold, motivated individuals) [38], though to our knowledge, our sample represents non-territorial, mostly juvenile caracaras.

### (b) Experimental protocol

**Figure 1.**
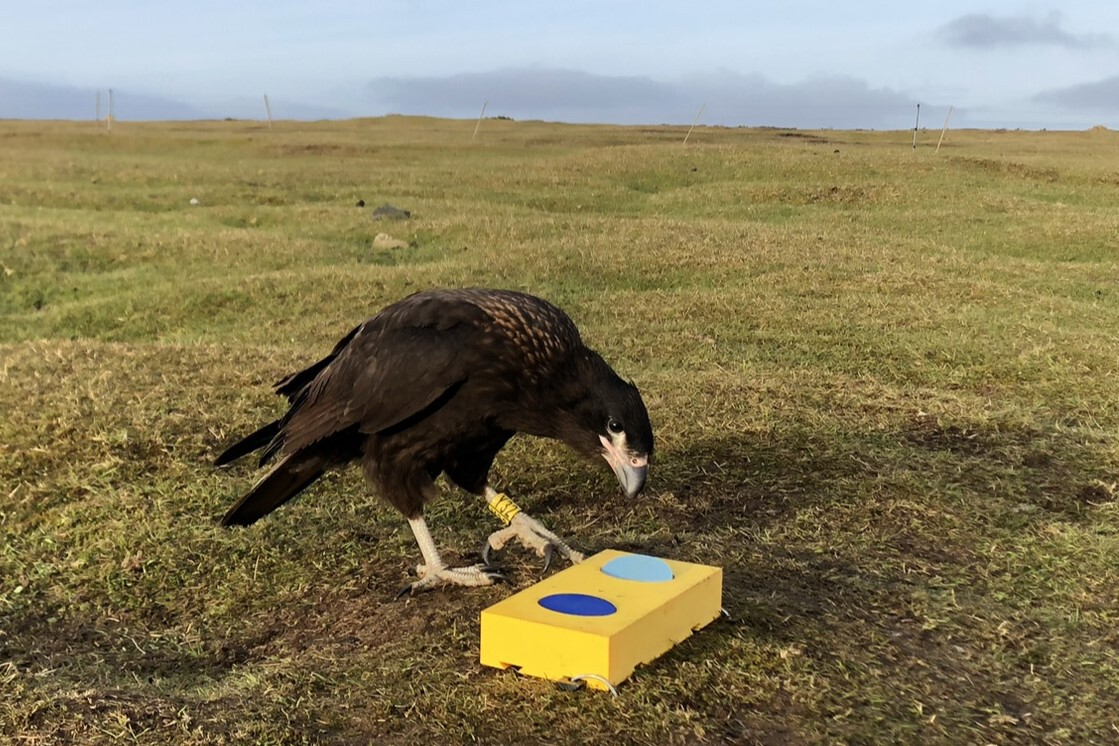
Wild striated caracara (*Phalcoboenus australis*) participating in food-rewarded colour discrimination task. Photo credit: Katie J. Harrington.

Testing followed a standardized field protocol [33]. Trials occurred on the ground, within a 3 m radial ‘trial arena’ marked by natural objects. The test apparatus was a yellow three-dimensional-printed foraging grid (20 × 5 × 3 cm; figure 1) containing two identical wells with opaque coloured lids that swivelled to reveal a hidden food reward (approx. 1 g mutton, a familiar, desirable food). Each well was regularly swabbed with the reward to control for odour cues. A trial began when a caracara voluntarily entered the arena and ended after first contact (‘choice’) with a lid; incorrect trials were unrewarded, with no second attempt. A brief training phase (≤3 rewarded trials with orange lids) familiarized birds with lid manipulation. For testing, we used light and dark blue lids, a colour unlikely to be naturally associated with food in their environment, to reduce potential colour bias or prior associations.

In the associative learning (AL) phase, each bird was pseudo-randomly assigned one rewarded colour (positive stimulus, S+) for the duration of trials, while the unrewarded colour well remained empty (negative stimulus, S−). Between trials, we visually blocked the apparatus, pseudo-randomly switched lid positions to mitigate potential side bias and rebaited the S+ colour well. Inter-trial intervals (approx. 20–30 s) imposed an opportunity cost. All trials were live coded as correct (first contact with S+) or incorrect (S−). Birds met the criterion by choosing S+ in 10 trials within a 12-trial running window. Total trials to reach the criterion was the AL score. Individuals who reached the criterion stopped AL testing to prevent overtraining, and all were limited to 50 trials per day (predetermined limit following [39]) to facilitate additional participants during limited daylight.

Individuals who reached the AL criterion became eligible for the RL task on their next available testing day (i.e. when next present at the study site; mean ± s.d. inter-task interval: 2.6 ± 2.5 days, range 1–11). We used the same procedure, but reversed reward contingencies (S+ became S−, and vice versa), requiring birds to inhibit the prior association. Birds could continue after reaching the criterion (limited to 50 trials per day) to allow performance assessment beyond TTC. TTC was recorded as the RL score. We used a single reversal, which, while limited relative to serial reversals in its ability to reveal broader flexibility (e.g. improvements across reversals, i.e. ‘learning to learn’) [7,23,40,41], nonetheless provides an effective measure of variation in flexibility under field constraints [15,42].

Task phases were run concurrently based on individual availability and task history.

### (c) Statistical analysis

We analysed 2406 trials from 32 individuals, 18 of which participated in both tasks (data from [43]). All analyses were conducted in R v. 4.3.3 [44].

To facilitate comparison with conventional metrics, we calculated TTC scores, overall accuracy (i.e. proportion correct), and for RL, post-criterion accuracy (proportion correct after reaching TTC).

To evaluate population-level learning trends while accounting for individual variation, we fitted a generalized linear mixed model with a binomial error structure and logit link function (glmer function, lme4 package, v. 1.1-32) [45]. The model predicted binary success as a function of trial number and task type; stimulus colour (control predictor); and the interaction of trial number and task type, which we included to test whether learning rates differed across AL and RL. Without this interaction, the model would assume a shared learning trajectory, which would be biologically implausible if the tasks engage distinct cognitive processes. We did not include age or sex as predictors, as previous work in a closely related species found no effect of age on RL [46], and our sample size was insufficient to support more complex models without risking spurious results or inadequate control for influential observations [47].

To account for repeated measures and individual differences in baseline accuracy and learning trajectories, we included random intercepts and possible random slopes per individual. Specifically, we fitted the maximal random slopes structure supported by the data (following [48,49]), including random slopes for the trial number × task interaction and colour. Prior to fitting, continuous covariates were *z*-transformed and categorical predictors were centred to improve convergence and interpretability of model coefficients. Model convergence was achieved using the bobyqa optimizer (200 000 iterations). We next verified key assumptions. We visually inspected histograms of the random effects (best linear unbiased predictors) using a custom diagnostic function (Roger Mundry, Leibniz ScienceCampus Primate Cognition, Germany), which indicated approximate normality. We assessed collinearity among predictors using the variance inflation factor (VIF) calculated from a linear model excluding the interaction and random effects (car package) [50]. VIF values were low (<1.2), indicating no issues with multicollinearity. We next assessed model stability by systematically excluding each level of the random effect using a custom function (Roger Mundry). Fixed effect estimates remained stable across reduced models, suggesting robustness to individual influence. To limit Type I error, we compared the full model to a null model excluding the effects of trial number and task using a likelihood ratio test [51]. We tested fixed effects using the drop1 function starting with the two-way interaction and continuing with main effects if this did not reveal significance. To ensure that model results were not biased by variation in trial count, we ran a trimmed version of the GLMM using the first 40 trials (maximum) per bird per task.

### (d) Individual learning models and null simulations

To assess individual learning, we fitted logistic regression models for each bird with a binomial error structure and logit link (glm function, lme4 package, v. 1.1-32 [45]). Each model predicted binary success as a function of trial number, yielding a slope coefficient on the log-odds scale that reflected direction and rate of learning.

To evaluate whether observed slopes exceeded chance, we simulated individual-specific null distributions. For each bird, we simulated 1000 random agents (*p* = 0.5) over the same number of trials, computed logistic slopes and calculated *p*-values as the proportion of null slopes ≥ observed. We considered slopes falling within the top 5% of the individual’s null distribution (*p* < 0.05) significant.

We evaluated individuals’ initial accuracy to detect potential early competence in AL and perseverative responses in RL, i.e. continued selection of the previously rewarded stimulus, which may have been masked by slope-based metrics. We computed individuals’ proportion correct in the first 12 trials and compared to a null distribution from 10 000 simulated agents (*p* = 0.5). We extracted the 5th and 95th percentiles to define thresholds for significantly below- and above-chance performance, respectively. We compared individuals’ overall accuracy in each task to chance, using two-sided binomial tests (*p* = 0.5).

We assessed cross-task consistency in individuals’ slopes using a Pearson correlation and tested whether the number of days between completing AL and beginning RL (i.e. inter-task interval) was related to RL performance (i.e. proportion correct) using Spearman’s rank correlation.

## 3. Results

Test predictors had a significant impact on the probability of success (full-null comparison: *χ*²_(2)_ = 23.15, *p* < 0.001, electronic supplementary material, table S1). However, the interaction was not significant (*χ*²_(1)_ = 0.09, *p* = 0.76), suggesting learning slopes did not differ between tasks; therefore, we removed it from the model to inspect the main effects.

At the group level, caracaras improved significantly across trials in both tasks, consistent with overall learning (reduced model: *β* = 0.512, s.e. = 0.103, *χ*²_(1)_ = 19.0, *p*  <  0.001, electronic supplementary material, table S1). Performance in RL was significantly lower than AL (reduced model: *β* = –1.00, s.e. = 0.20, *χ*²_(1)_ = 15.97, *p*  <  0.001, electronic supplementary material, table S1), in line with the additional demands of reversing a previously learnt association. This was also reflected in overall accuracy, which was lower in RL (mean correct: 0.48 ± 0.14 s.d.) than in AL (0.66 ± 0.17). A modest colour effect favoured dark blue (*β* = –0.83, s.e. = 0.16, *χ*²_(1)_ = 24.106, *p*  <  0.001, electronic supplementary material, table S1). Fixed effect estimates from the full and trimmed models were qualitatively consistent, and random effect variances were comparable, suggesting that learning and task effects were robust to variation in trial count (electronic supplementary material, table S1).

**Figure 2.**
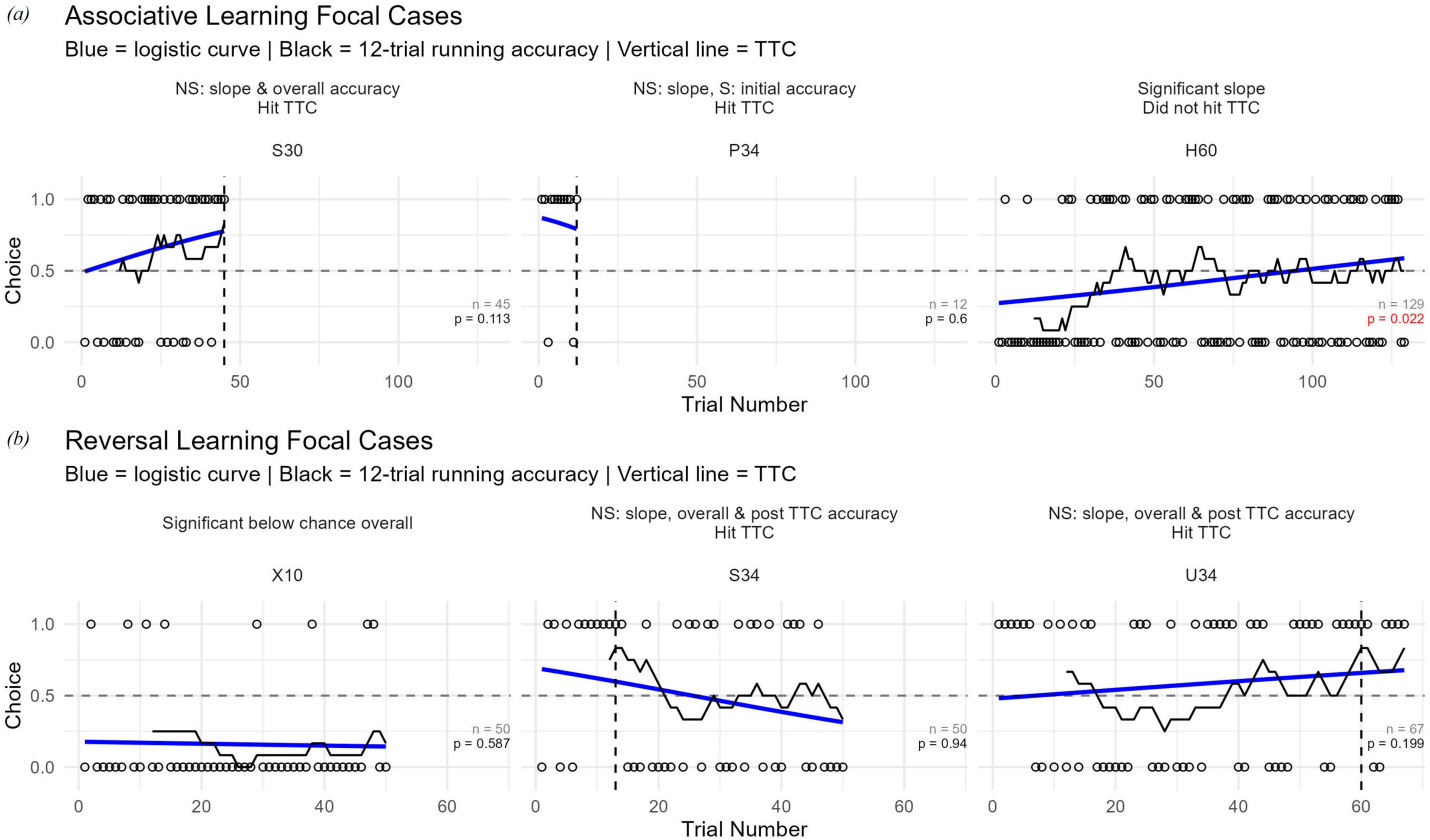
Focal cases from AL (*a*) and RL (*b*) tasks illustrating limitations of TTC. Open circles show choices across trials (1 = correct, 0 = incorrect). Lines indicate 12-trial running accuracy (black) and logistic learning curves (blue). Vertical lines mark TTC. *p*-Values reflect the proportion of null slopes ≥ the observed slope (red: *p* < 0.05). Sample size (*n*) is total trials completed. Horizontal dashed line illustrates chance. Plot labels highlight inconsistent metrics (i.e. non-significant (NS) or significant (S) slope, initial accuracy, overall accuracy and post-criterion accuracy; and when TTC was met).

We next assessed individual learning. In the associative phase, 10/32 birds showed significantly positive learning trajectories relative to chance simulations (hereafter LC learners; table 1, electronic supplementary material, figure S1). An additional eight birds showed significantly high initial accuracy (0.87 ± 0.06; table 1, figure 2*a*), but non-significant slopes due to few trials. In contrast, 25/32 met the criterion (hereafter TTC birds; AL score: 27 ± 16, range 10–65 trials), including 16 with non-significant slopes and missing one bird with a significant slope (figure 2*a*). Of TTC birds, 13 were above-chance overall (0.82 ± 0.09); 12 were not (0.59 ± 0.06).

**Table 1.** Participant summary by task, including total trials (*n*); TTC; results compared to chance (i.e. above, not significant (NS) or below) for initial accuracy (first 12 trials), overall accuracy (proportion correct), slope and RL post-criterion accuracy (Post-TTC); and RL post-criterion total trials (Post-*n*). Above chance slopes indicate slope-based learning. Note the five potential non-learners (i.e. NS AL slopes) advanced to the reversal stage whose above chance RL slopes likely indicate initial rather than reversal learning.

	associative learning task					reversal learning task						
ID	*n*	TTC	initial accuracy	overall accuracy	slope	*n*	TTC	initial accuracy	overall accuracy	Post-TTC	Post-*n*	slope
S34	21	21	NS	NS	above	50	13	NS	NS	NS	37	NS
U34	48	48	NS	NS	above	67	60	NS	NS	NS	7	NS
R34	20	20	NS	NS	above	65	48	NS	NS	NS	17	above
V11	22	22	NS	NS	above	100	85	below	below	NS	15	above
Y14	42	38	NS	NS	above	74	67	NS	NS	NS	7	above
K33	66	65	below	NS	above	70	68	NS	NS	NS	2	above
V34	12	12	above	above	above	—	—	—	—	—	—	—
S33	37	37	NS	NS	above	—	—	—	—	—	—	—
U13	43	43	NS	NS	above	—	—	—	—	—	—	—
H60	129	—	below	NS	above	—	—	—	—	—	—	—
U11	12	12	above	above	NS	39	28	NS	NS	NS	11	NS
G60	12	12	above	above	NS	74	—	below	below	—	—	NS
W12	11	11	above	above	NS	50	45	NS	NS	NS	5	NS
X10	53	52	NS	above	NS	50	—	NS	below	—	—	NS
B34	17	17	NS	above	NS	9	—	below	NS	—	—	NS
P34	12	12	above	above	NS	50	45	NS	NS	NS	5	NS
V32	33	33	NS	NS	NS	135	—	NS	NS	—	—	NS
Y34	18	12	above	above	NS	136	122	below	NS	NS	14	above
P19	47	47	NS	above	NS	50	42	below	NS	NS	8	above
B35	10	10	above	above	NS	62	61	NS	NS	NS	1	above
Y32	12	12	above	above	NS	101	101	below	NS	—	—	above
K35	21	21	NS	NS	NS	77	55	NS	above	above	22	above
S16	11	11	above	above	NS	—	—	—	—	—	—	—
W11	42	30	NS	above	NS	—	—	—	—	—	—	—
B11	29	28	NS	NS	NS	—	—	—	—	—	—	—
S30	45	45	NS	NS	NS	—	—	—	—	—	—	—
G11	9	—	below	NS	NS	—	—	—	—	—	—	—
Z12	73	—	NS	NS	NS	—	—	—	—	—	—	—
E18	130	—	NS	NS	NS	—	—	—	—	—	—	—
R35	96	—	NS	NS	NS	—	—	—	—	—	—	—
Y33	10	—	NS	NS	NS	—	—	—	—	—	—	—
U35	4	—	NS	NS	NS	—	—	—	—	—	—	—

In the reversal phase, among LC learners with RL data (*n* = 6), four showed significant reversal trajectories (LC reversers; table 1, electronic supplementary material, figure S2), including one with below-chance initial accuracy (i.e. perseverative error). Among TTC birds with RL data (*n* = 18), 14 reached the reversal threshold (RL score: 60 ± 28, range 13–122). Five of the 14 had non-significant slopes and were not significantly different from chance in initial nor post-criterion accuracies (table 1, figure 2*b*). Of the remaining nine TTC birds with significant slopes, four began below chance, including one with a below average overall accuracy, while one maintained above-chance overall and post-criterion accuracy (table 1, figure 2*b*). Twelve TTC birds had overall performances that did not differ from chance (0.54 ± 0.06; table 1, figure 2*b*).

Learning slopes across tasks were not correlated (*r* = −0.08, *p* = 0.75) nor were inter-task interval and RL performance (*r* = 0.05, *p* = 0.83).

## 4. Discussion

Striated caracaras showed evidence of learning across both tasks, although reversing appeared to be more difficult, with early perseverative errors and lower overall accuracy. Despite this, many caracaras showed positive RL slopes, suggesting flexible updating was possible—an ability likely shaped by the ecological demands of coping with seasonal changes in food availability [25,52,53].

### (a) Beyond trials-to-criterion

Our results underscore the interpretive limits of TTC [10–12,20]. In the AL task, TTC identified 25 learners—150% more than the 10 birds identified by significant learning slopes—suggesting many individuals were advanced to reversal without acquiring the initial association (figure 2). Some of these cases were driven by high initial accuracy, but because TTC imposes a sharp cut-off, it cannot distinguish genuine acquisition from early chance streaks and thus provides no evidence of sustained learning. Conversely, TTC overlooked a bird (H60) that shed an initial colour bias (S−) and then performed at chance (i.e. significant learning slope, though no acquisition; figure 2*a*). In the RL task, five of the TTC birds showed no evidence of RL (i.e. non-significant slopes; figure 2*b*), despite reaching criteria, while five others produced significant reversal slopes likely reflecting initial acquisition (table 1). At the group level, TTC may sometimes correlate moderately with overall accuracy under stringent conditions, but validation by supporting metrics such as post-criterion accuracy or trajectory-based modelling is essential [20].

Among birds with slope-based evidence of learning (LC learners), TTC misclassified reversal performance. For example, S34 met reversal criteria by trial 13 but then declined, suggesting an early performance spike rather than stable acquisition, while U34 reached the criterion after 60 trials despite a non-significant slope (figure 2*b*). By reducing learning to a threshold, TTC cannot assess whether performance is maintained or distinguish persistence from competence. More broadly, any paradigm that reduces learning to a discrete threshold—whether binary, multi-choice or spatial tasks—faces the same limitations. In contrast, trajectory-based approaches model performance across all trials, remain robust to variation in trial number (electronic supplementary material, table S1) and provide a more reliable framework for evaluating learning [20].

The probability of meeting the criterion exactly at individuals’ observed TTC is small, although the probability of doing so at any point up to then is non-trivial because of repeated sequential opportunities, highlighting TTC’s vulnerability to false positives (electronic supplementary material, table S2). In contrast, random agents rarely produced the systematic positive slopes observed in true learners. These analyses therefore provide a more conservative and biologically meaningful benchmark, underscoring the need to move beyond TTC towards trajectory-based approaches.

### (b) Motivation, persistence and behavioural noise

Individuals performing at or below chance cannot be assumed to have failed to learn, as they may have adopted alternative strategies, engaged variably with the task depending on motivational state or exploited cues not detectable in our design [13]. Caracaras often continued engaging after repeated incorrect (i.e. unrewarded) trials, which suggests intrinsically rewarding engagement, e.g. tactile exploration [54]. Such behavioural momentum can sustain interaction but may dilute the salience of negative feedback, weakening extinction or slowing contingency updates.

High engagement despite incorrect choices may reflect exploratory bias, whereby individuals continue sampling unrewarded options despite accumulated evidence—consistent with undirected exploration strategies and the ‘information primacy’ model [55,56], which may be adaptive in environments where the value of options can shift over time [24]. In binary tasks, however, such patterns are difficult to disentangle from motivational state: they could reflect reduced motivation for the food reward, rather than directed sampling. In our case, high participation suggests strong task motivation despite low food motivation. Furthermore, if the task is sufficiently simple, individuals may rely on heuristics (e.g. win–stay/lose–shift) or random sampling, yielding streaks of correct or incorrect choices without evidence of learning [57]. Engagement may also be facilitated when unrewarded trials carry little cost—such as minimal inter-trial delay or energetic penalty—which can decouple engagement from accuracy and mask cognitive flexibility. Alternatively, sustained effort may lead to decision fatigue and increased choice noise over time [58]; or the task apparatus may have acquired a globally reinforcing value, akin to a regenerating foraging site, encouraging generalized win–stay strategies not tied to stimulus cues [59]. This points to the complex dynamics underlying task performance and further cautions against reductive metrics such as TTC as a proxy for learning.

### (c) Task design considerations

Experimental design may have influenced our ability to detect other learning strategies. While variable approach angles minimized the likelihood of fixed spatial cue use, they also obscured detection of whether individuals attempted alternative spatial heuristics (e.g. choices linked to approach path) [59,60]. Because such rules can take many forms in natural environments, incorporating them would have introduced more predictors than our sample size could support. Additionally, the colour stimuli—dark and light blue—may have elicited bias by being too perceptually distinct. Future designs should consider validating species-specific cue discriminability in advance [9] and balance cue randomization with controlled approach paths to increase transparency of individual decision-making while maintaining ecological validity.

### (d) Dissociable performance across tasks

Slopes were uncorrelated across tasks, consistent with research suggesting the tasks engage distinct cognitive processes [46,60,61]. AL performance may be shaped more by perceptual salience, initial attention or colour preference, whereas RL—consistent with its role as a benchmark for behavioural flexibility—requires greater sensitivity to contingency changes. The colour effect in AL but not RL supports the view that these tasks rely on partly distinct mechanisms.

### (e) Redefining learning in the wild

Our results argue for more nuanced approaches to measuring learning, particularly in field contexts where trial counts can vary widely due to access, motivation or social factors. TTC risks misclassifying individuals by failing to distinguish stochastic success from stable behavioural change. Slope-based analyses offer a continuous, comparable metric that reduces methodological artefacts and improves cross-study inference [9]. Given the widespread continued use of TTC as a proxy for cognitive ability in animal cognition research [62–64], this has important implications for how learning and competence are inferred across individuals, contexts and species.

## Data Availability

Our data was live coded by hand and later entered into a CSV file. The CSV file contains individuals' binary response across trials per task. Variables are defined as following: date (mm/dd/Y), ID (individual), age (HY, hatch-year; JUV, juvenile; SA, sub-adult; AD, adult; identified by plumage), sex (F, female; M, male; identified by mass), task (AL, associative learning; RL, reversal learning), colour (LB, light blue; DB, dark blue), session (set of trials corresponding to a single day), trial (trial number), succ (0, incorrect; 1, correct), cumsum (cumulative sum of correct choices), cumTrial (cumulative trial number, i.e. pooled across sessions), crit (0, no; 1, yes, i.e. whether an individual has met the criterion by that trial number) and critTrial (trial in which individual first reached the criterion). Our R script for analysing the data is fully annotated. Our data and R script are available here: [43]. Supplementary material is available online [65].
